# Feather colour affects the aggressive behaviour of chickens with the same genotype on the dominant white (*I*) locus

**DOI:** 10.1371/journal.pone.0215921

**Published:** 2019-05-02

**Authors:** Changsheng Nie, Liping Ban, Zhonghua Ning, Lujiang Qu

**Affiliations:** 1 State Key Laboratory of Animal Nutrition, Department of Animal Genetics and Breeding, National Engineering Laboratory for Animal Breeding, College of Animal Science and Technology, China Agricultural University, Beijing, China; 2 College of grassland science and technology, China Agricultural University, Beijing, China; Tokat Gaziosmanpasa University, TURKEY

## Abstract

Aggression in chickens is a serious economic and animal welfare issue in poultry farming. Pigmentation traits have been documented to be associated with animal behaviour. Chicken pecking behaviour has been found to be related to feather colour, with premelanosome protein 17 (*PMEL17*) being one of the candidate genes. In the present study, we performed a genotypic and phenotypic association analysis between chicken plumage colour (red and white) and aggressive behaviour in an F1 hybrid group generated by crossing the autosomal dominant white-feathered breed White Leghorn (WL) and the red-feathered breed Rhode Island Red (RIR). In genetic theory, all the progeny should have white feathers because WL is homozygous autosomal dominant for white feathers. However, we found a few red-feathered female chickens. We compared the aggressiveness between the red and white females to determine whether the feather colour alone affected the behaviour, given that the genetic background should be the same except for feather colour. The aggressiveness was recorded 5 days after sexual maturity at 26 weeks. Generally, white plumage hens showed significantly higher aggressiveness compared to the red ones in chasing, attacking, pecking, and threatening behaviour traits. We assessed three candidate feather colour genes—*PMEL17*, solute carrier family 45 member 2 (*SLC45A2*), and SRY-box 10 (*SOX10*)—to determine the genetic basis for the red and white feather colour in our hybrid population; there was no association between the three loci and feather colour. The distinct behavioural findings observed herein provide clues to the mechanisms underlying the association between phenotype and behaviour in chickens. We suggest that mixing red and white chickens together might reduce the occurrence of aggressive behaviours.

## Introduction

Aggressive behaviour in chickens is a widespread economic and animal welfare issue in poultry farming, and many factors such as food, mates, and social rank affect its occurrence [1, 2]. Such behaviour has different forms (threats and intense agonistic behaviour) and is divided into numerous types such as still threats, chasing, aggressive pecking, and attacks [3]. As a complex trait in chicken, aggressiveness has been found to be present, albeit with low heritability (h^2^ = 0.04–0.17) in various studies [4–8], and aggressive pecking has been reported to be affected by a variety of environmental factors, including light intensity [9], stocking density [10, 11], food [12], feeding methods [13], group size [14], and male presence [1]. Moreover, appearance factors such as comb type [15], plumage pattern [16], and plumage colour [17] can also influence the behaviour of chickens. Therefore, aggressive behaviour in chickens is a function of interaction among genes, phenotype, and environment.

In terms of the genetics of aggressiveness, a few candidate genes, including premelanosome protein 17 (*PMEL17*, *I* locus), solute carrier family 45 member 2 (*SLC12A9*), G protein subunit gamma 2 (*GNG2*), calsyntenin-2 (*CLSTN2*), brain-derived neurotrophic factor (*BDNF*), neurotensin (*NTS*), G protein subunit alpha o1 (*GNAO1*), and sortilin-related VPS10 domain-containing receptor 2 (*SORCS2*) have been identified to be correlated to the trait [18–20]. As a domesticated trait, colour phenotypes may also be selected as side-effects linked to behaviour during domestication [17]. Previous studies on pigs [21], silver foxes [22], Norway rats [23], deer mice [24], mink [25], and chickens strongly supported this point. In chickens, feather-pecking damage had a highly significant QTL that coincided perfectly with the dominant white locus (*I*) [26], and the *PMEL17* gene was found to be responsible for this phenotype [27]. Moreover, the *PMEL17* locus could also affect explorative and social behaviours in chickens [17].

However, whether *PMEL17* is directly associated with behaviour or indirectly influences feather colour remains unknown. If the *PMEL17* locus were consistent (*Ii*), whether there are other factors that might affect chickens’ feather colour and behaviour and how long the impact can last also remain subject to investigation. In this study, we used individuals with two different feather colours (white and red) and controlled the effect of the *I* locus to discover the behavioural diversity in mature chickens.

## Materials and methods

All procedures and protocols (DOI: dx.doi.org/10.17504/protocols.io.ysgfwbw) involving animals were conducted in accordance with the Guidelines for the Care and Use of Experimental Animals established by the Ministry of Agriculture of China (Beijing, China). All the animal protocols were approved by the Animal Welfare Committee of China Agricultural University (Beijing, China, Permit Number: XK622).

### Animals

The chickens used were the offspring of a cross between RIR (male) and WL (female). WL’s dominant white plumage and RIR’s sex-linked recessive red plumage were genetically determined by *PMEL17* on an autosome (denoted as *I*) and *SLC45A2* on a Z chromosome (denoted as *Z*^*s*^), respectively. The genotypes of the two loci in WL are *IIZ*^*S*^*Z*^*S*^*/IIZ*^*S*^*W*, and for RIR, they are *iiZ*^*s*^*Z*^*s*^*/is* for male/female. Since white feathers in WL are autosomal dominant homozygous (*II*), the daughters or sons of WL are expected to have white plumage (*I-*). However, when we crossed female WL (*II*) and male RIR (*Z*^*s*^*Z*^*s*^), some exceptions were observed, with a few daughters (*IiZ*^*s*^*W*) presenting with red feathers or red-coloured heads, although most of the daughters (*IiZ*^*s*^*W*) were white. The genetic basis underlying these exceptions remains to be identified.

The WL and RIR chickens used in this study were maintained by a commercial company (Hehei Dawu Co., Ltd.) and had undergone selection for production traits for more than 30 generations. We believed that the *I* and *Z*^*s*^ locus of the F1 females ought to be the same; we separated the birds into those with white feathers, red feathers, and red-coloured heads. Therefore, these groups of birds formed an ideal population for investigating the association, without PMEL17’s influence, between pigmentation and the behaviour of chickens.

**Fig 1:**
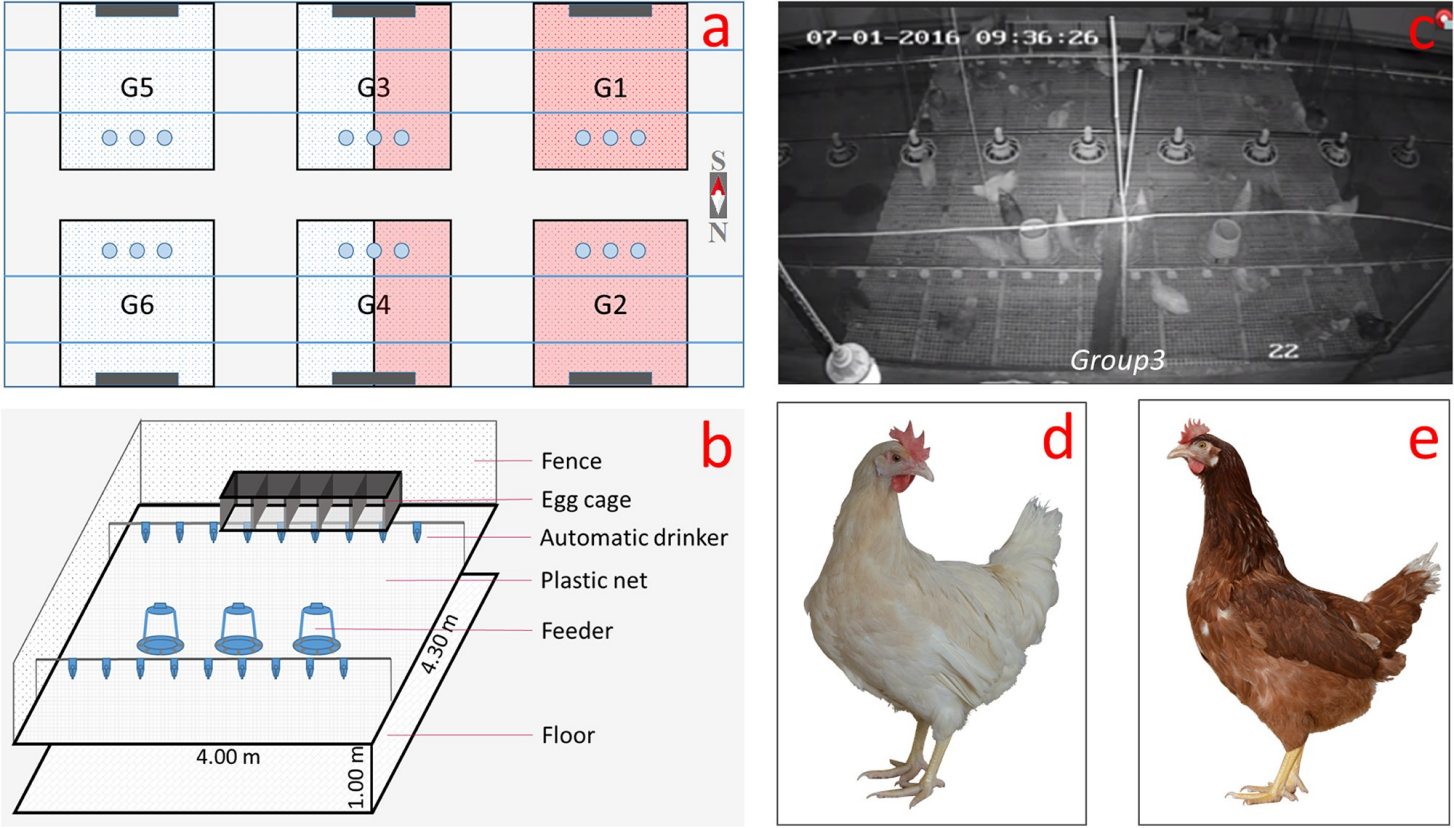
Illustration of the housing conditions and animal phenotypes. (a) Six pens in the facility. G1-6 represents groups 1–6, respectively. (b) Internal structure of the pen. (c) Monitor recording animal behaviours (Group 3). (d) White and (e) red feathered chickens.

We crossed male RIR and female WL to obtain F1 hybrids (commercial line) and 150 red-feathered daughters, and 150 white-feathered daughters (Fig 1) were randomly selected for our experiments. All the hens were examined in further behavioural tests.

### Housing conditions of animals

The eggs from the cross between RIR males and WL females were hatched at the same time. We collected red- and white-feathered female chickens (because all the males were white). We reared the selected females in the same chicken house, where the lighting, temperature, and ventilation were completely artificially controlled. The temperature was maintained at 23°C with ventilation fans and cooling pads. The chickens were housed in six pens with a plastic net ground (4.30 m long × 4.00 m width × 2.00 m high, 1.00 m above the floor). The pens were equipped with automatic drinkers (Fig 1), and clean water (fresh) was available *ad libitum* to the birds. The chickens were fed manually with standard commercial chicken feed. No adverse effects such as severe exhaustion, illness, or severe injury were observed to have resulted from the experiment. In addition, the birds were exposed to a 13.5L:10.5D photoperiod (from 0700 to 2030 hours) with a light power of 5 W (26 weeks).

### Behavioural tests

Red and white hens were separately reared in chick cages (~30 chickens/cage) from 1–115 days and transferred to adult chicken cages (3 chickens /cage) after 115 days. At 20 weeks old, the chickens were transferred to the pens and were divided into six groups of 50 birds each.

Groups 1 and 2 were replicates for the red-feathered hens, while groups 3 and 4 were the same, each containing 25 red and 25 white hens. Groups 5 and 6 were replicates for the white hens (Fig 1). All the birds were allowed to adapt to the new conditions for 5 weeks. After the adaptation period, aggressive behaviours were recorded using a monitor from day 177 to 181 (26 weeks), since the females were sexually mature and tended to be more aggressive at this time than before attaining sexual maturity. Moreover, body weight was recorded at the beginning (20 weeks) and end (25 weeks) of the adaptation period.

Behaviour was recorded between 1200 and 1230 hours by one person using the same standard (30 min × 5 days × 6 pens; S1 Table). Two main behavioural features, B1 and B2, were recorded in this study. B1 consisted of the chase and attack behaviour, while B2 comprised the peck and threat behaviour (Table 1). These behaviours were counted separately (attacker or victim) for the red- and white-feathered hens in the blended group (groups 3 and 4). The behavioural definitions were based on the ethogram of Väisänen [3]. Each distinct behaviour pattern occurring with the two same hens (attacker or victim) was counted as one event independent of its duration unless suspended by any other behaviour for more than 3 s. Due to the inability to identify individuals within a pen, we used the entire pen’s behavioural occurrence time as the standard unit.

**Table 1:** Two aggressive behaviour phenotypes in female chickens demonstrated in this study.

Abbreviation	Categories	Behaviour description [3]
**B1**	**Chase**	Bird follows another; both birds run, jump, or fly.
	**Attack**	Bird jumps, flies, runs, or takes fast steps when approaching another bird to give it an aggressive peck; birds stand or walk >1 m away from each other.
**B2**	**Peck**	Bird rapidly pecks the anterior part of another bird.
	**Threat**	Stiff body posture towards another bird; the birds stand <0.25 m from each other. The head is positioned above or below the receiver’s head. Feathers may be lifted.

### Genotyping candidate genes for feather colour

To delineate the genotypes underlying the red and white feather colour and determine if they directly or indirectly influence behaviour, we selected locus 3 as the gene for genotyping and association analysis. Since we already knew the genotype of locus 2, including dominant white feathers (*I*) and sex-linked recessive red feathers (*Z*^*s*^), we picked the SRY-box 10 (*SOX10*) gene for genotyping. *SOX10* is responsible for the dark brown (*DB*) phenotype, which is caused by an 8.3 kb deletion upstream of the transcription start site [28].

A total of 60 females were selected, consisting of 29 and 31 with white- and red-feathered birds, respectively, from the F1 population. Wing vein blood was obtained from red- and white-feathered hens (without anaesthetic). Genomic DNA was extracted using standard phenol/chloroform protocols. The DNA concentration was determined using a NanoDrop 2000 spectrophotometer (Thermo Fisher Scientific Inc.).

Polymerase chain reactions (PCRs) were performed using a Veriti 96 Well thermal cycler (Applied Biosystems) according to the manufacturer’s protocol. The polymorph of the 8.3 kb deletion upstream of the *SOX10* gene was amplified using PCR with the 1 forward (1F, 5′-CCTTTGTCTTAAGGCTCCTCTTT-3′), 1 reverse (1R, 5′-CCTTGTGGAGACCAGGTGTT-3′), and 6R (5′-TGCTGAGACATTTGCTGACA-3′) primers from Gunnarsson et al. [28]. Fragments of 611 (1F/1R) and 1257 bp (1F/6R) associated with the wild-type (*db/db*) and dark brown (*DB*/DB) alleles, respectively, were displayed by agarose gel electrophoresis.

### Statistical methods

The descriptive statistics were analysed using the Statistical Package for the Social Sciences (SPSS) software using all available records [29]. The general linear model was used for analysing the main and interactive effects of feather colour and position (south or north; Fig 1) on the occurrence of behaviours. In addition, Tukey’s honestly significant difference test was conducted for pairwise comparisons (behaviours and body weight) between different groups.

## Results

### Body weight

In the present study, hens with two feather colours obtained from the cross were raised in the same facility under the same environmental conditions. To minimize body size differences, all chickens were randomly separated to six pans (20 weeks). Although body weight differed (G1 and G2 were significantly heavier than G3 and G5) in six groups at the age of 20 weeks (Table 2), no significant difference in body weight was found in the repeat groups (G1 vs. G2, G3 vs. G4, and G5 vs. G6). After 5 weeks of adaptation, there was no significant difference among the feeding pans of the three types (red, mingled, and white groups) (Table 2).

**Table 2:** Descriptive statistics of body weight (20 and 25 weeks) in six groups.

Feather colour		Red		Mingled		White	
Group		G1 (n = 50)	G2 (n = 50)	G3 (n = 50)	G4 (n = 50)	G5 (n = 50)	G6 (n = 50)
**Body weight (20 wks)**	**Mean ± SD (g)**	1434.10 ± 110.80^a^	1426.92 ± 105.37^a^	1354.22 ± 112.96^b^	1367.06 ± 125.68^ab^	1343.40 ± 116.08^b^	1388.06 ± 132.95^ab^
	**CV (%)**	7.73	7.38	8.34	9.19	8.64	9.58
**Body weight (25 wks)**	**Mean ± SD (g)**	1556.22 ± 132.75	1570.62 ± 101.37	1531.64 ± 129.63	1530.44 ± 101.10	1535.34 ± 98.59	1562.82 ± 94.29
	**CV (%)**	8.53	6.45	8.46	6.61	6.42	6.03

However, the mingled group showed an inconsistent result. There was no difference between the body weight of red- and white-feathered birds (group 3), but it differed after adaptation (Table 3). Thus, most individuals had a relatively uniform size in the behaviour tests.

**Table 3:** Descriptive statistics of body weight (20 and 25 weeks) in mingled groups (group 3 and group 4).

Mingled group		Group 3		Group 4	
Feather colour		White (n = 25)	Red (n = 25)	White (n = 25)	Red (n = 25)
**Body weight (20 wks)**	**Mean ± SD (g)**	1348.52 ± 115.02	1359.92 ± 112.94	1387.00 ± 130.32	1347.12 ± 120.16
	**CV (%)**	8.53	8.31	9.40	8.92
**Body weight (25 wks)**	**Mean ± SD (g)**	1586.40 ± 103.81^a^	1476.88 ± 131.46^b^	1536.56 ± 97.72^ab^	1524.32 ± 106.02^ab^
	**CV (%)**	6.54	8.90	6.36	6.96

### Aggressive behaviours

**Fig 2:**
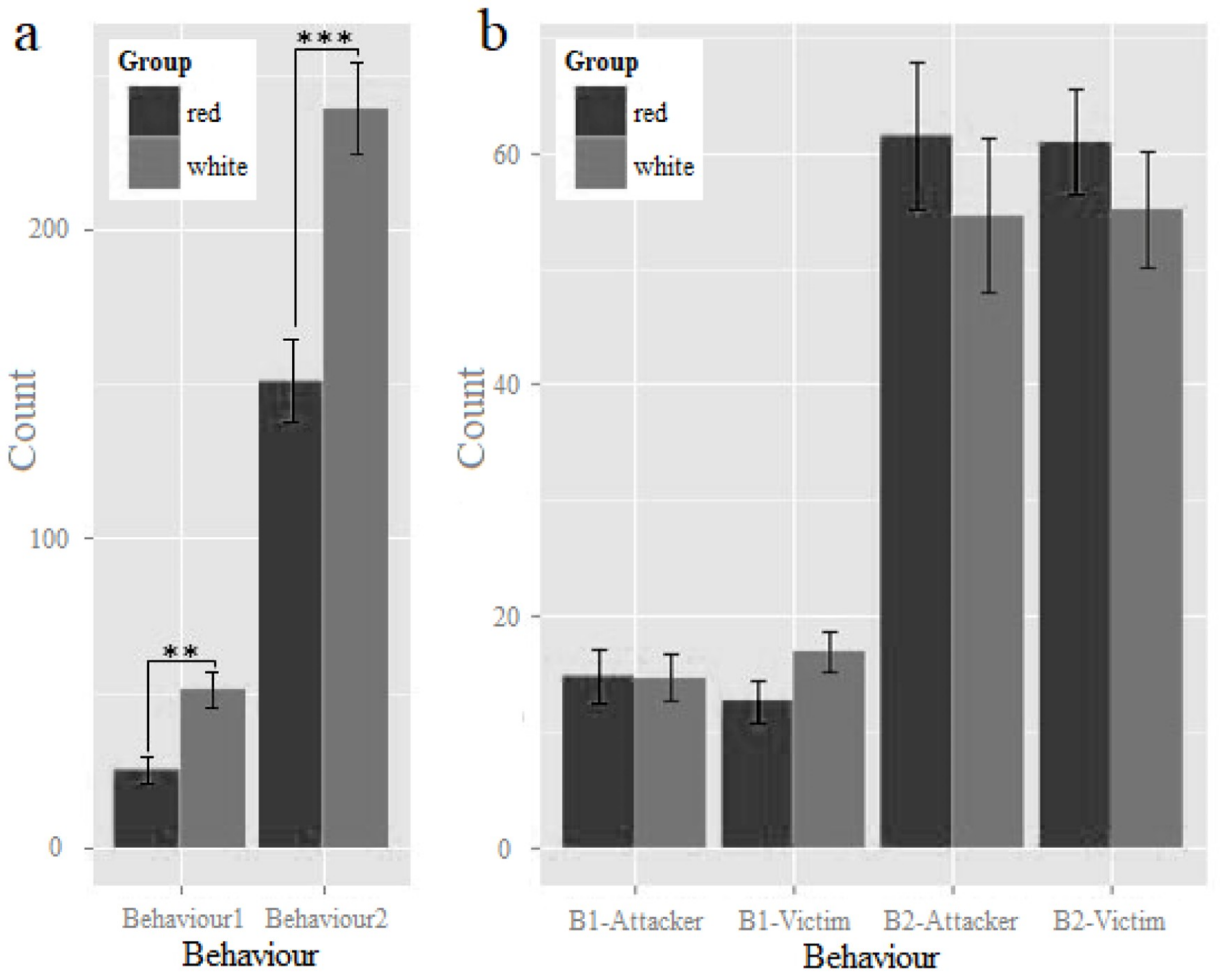
Aggressive behaviours count in different feather colours hens. (a) Red and white feather coloured groups (mean ± SE). (b) Mingled group (mean ± SE).

All birds were healthy throughout the observation period, and we easily recorded their aggressive behaviours. Feather colour had a significant effect on the counts of both B1 and B2 aggressive behavioural traits measured (Table 4). The white hen group (G5 and G6) exhibited significantly (p < 0.001) higher aggressive levels of chase, attack, peck, and threat behaviours than the red hen group (G1 and G2) did (Fig 2). Moreover, the mean number of aggressive behaviours exhibited was lower among the mingled groups than the white groups (29.50 vs. 50.90 in B1, 116.10 vs. 239.60 in B2). Pairwise comparison between the red and mingled group did not reveal significant differences in counts of B1 and B2.

**Table 4:** P- and F-values for the main effects of feather colour (red, mingled, and white), position (north and south), as well as the interactive effect of feather colour × position on the behavioural variables obtained by GLM.

Variable	Feather colour		Position		Feather colour × position	
	P	F	P	F	P	F
**Behaviour 1**	<0.001	10.825	0.093	3.055	0.205	1.696
**Behaviour 2**	<0.001	23.668	0.882	0.023	0.748	0.294

The results of each group are shown in Tables 5 and 6. Peck and threat behaviours (109.40 to 242.60) occurred more frequently than chase and fight (24.80 to 61.20) did in the three different types of hen groups. Behavioural traits were highly variable, and descriptive statistics of behaviours in each group showed very high coefficient of variation (14.16% to 59.09%) (Table 5).

**Table 5:** Descriptive statistics of two aggressive behaviours in six groups (5 days).

Feather colour		Red		Mingled		White	
Group		G1 (n = 5)	G2 (n = 5)	G3 (n = 5)	G4 (n = 5)	G5 (n = 5)	G6 (n = 5)
**Behaviour 1 (chase, attack)**	**Mean ± SD**	24.80 ± 12.89	25.40 ± 15.01	32.20 ± 9.63	26.80 ± 7.98	61.20 ± 21.49	40.60 ± 6.80
	**CV (%)**	51.98	59.09	29.9	29.78	35.11	16.76
**Behaviour 2** **(peck, threat)**	**Mean ± SD**	158.40 ± 45.57	144.20 ± 41.38	109.40 ± 33.35	122.80 ± 22.83	242.60 ± 60.97	236.60 ± 33.49
	**CV (%)**	28.77	28.7	30.49	18.59	25.13	14.16

**Table 6:** Descriptive statistics of two aggressive behaviours in mingled groups (5 days).

Group		Group 3		Group 4	
Feather colour		White (n = 5)	Red (n = 5)	White (n = 5)	Red (n = 5)
**B1_attacker**	**Mean ± SD**	12.00 ± 3.74	20.20 ± 6.50	17.40 ± 7.27	9.40 ± 2.79
	**CV (%)**	31.18	32.16	41.76	29.71
**B2_attacker**	**Mean ± SD**	39.60 ± 11.15	69.80 ± 24.10	69.60 ± 17.46	53.20 ± 12.28
	**CV (%)**	28.15	34.52	25.08	23.08
**B1_victim**	**Mean ± SD**	16.40 ± 6.66	15.80 ± 5.81	17.40 ± 4.22	9.40 ± 4.72
	**CV (%)**	40.58	36.74	24.25	50.24
**B2_victim**	**Mean ± SD**	54.00 ± 18.34	55.40 ± 15.87	56.20 ± 14.79	66.60 ± 11.17
	**CV (%)**	33.97	28.64	26.31	16.77

Furthermore, we counted the number of attacks and victim experiences between the red and white hens in the mingled groups, respectively (Table 6). However, there was no significant difference for B1-attacker, B2-attacker, B1-victim, or B2-victim between the red and white hens (Fig 2).

### Genotyping

The genotype of the *SOX10* deletion for both red and white plumage hens is shown in Table 7. The homozygous *DB* deletion did not appear in any of the tested hens. Two types of *SOX10* genotypes were recognised—heterozygous *DB* deletion and non-deleted wild-type—in both the red and white plumage hens, indicating that this locus was not linked to feather colour phenotypes.

**Table 7:** Plumage colour and related genotype distribution in the F1 generation of the White Leghorn/Rhode Island Red cross.

Phenotype	*SOX10*		
	*DB/DB*^1^	*DB/db*^2^	*db/db*^3^
**Red**	0	15	14
**White**	0	23	8

^***1***^*DB/DB*: dark brown allele

^***2***^*DB/db*: heterozygote allele

^***3***^*db/db*: wild-type allele.

## Discussion

The domestication of animals is associated with feather or coat colour changes in various animal species [22, 30]. In mammals, these observations suggest that domestication has a co-evolutionary effect of inducing pigment loss and tame behaviour, which has also been proven in birds.

In the present study, the birds used were the offspring of the first generation of a cross between RIR (*iiZ*^*s*^*Z*^*s*^) and WL (*IIZ*^*S*^*W*). However, a few daughters (*IiZ*^*s*^*W*) presented with red feathers or red-coloured heads, although most were white (*IiZ*^*s*^*W*). The emergence of red-feathered hens shows that the epistatic effect of *PMEL17* on *SLC45A2* was reduced. Moreover, behavioural observations of the red (groups 1 and 2) and white (groups 5 and 6) groups indicated that white plumage hens were more aggressive than the coloured hens were, although all the birds had the same *I* locus (*I/i)*.

Keeling (2009) showed that feather damage due to feather-pecking by other birds was 3.34 times higher in pigmented birds (*i/i*) than that in white (*I/I*) birds. However, the tendency for red (pigment type, *I/i*) birds to experience more attacks compared to white birds (*I/i*) was not significant in this study (Fig 2), which might be explained in three possible ways. First, the same genotype (*I/i*) might lead to uniform behavioural effects. Second, a huge coefficient of variation might reduce the difference between red and white hens in the mingled groups (group 3 and group 4). Third, the small sample size (n = 25) of the groups might have affected the results.

From the perspective of genetics, one or more genes might be controlling the different plumage colour formation in the present population. Unfortunately, consistent with the *SOX10* genotyping results, neither *SOX10* nor *PMEL17* caused the red/white phenotype or produced dramatically different behavioural changes in the present population [17, 26]. Meanwhile, it is still unclear how feather colours and pigments affect (directly or indirectly) each other.

Normally, two main pigments in birds, eumelanin and pheomelanin, are generated from l-tyrosine after a series of reactions [31], and dopa and dopa analogues are crucial mid products [32]. In addition, the serotonergic and dopaminergic systems also have possible roles in the aetiology of feather pecking [33–36]. *DRD4*, encoding the dopamine receptor D4, has been determined to be associated with the behaviour of birds [37]. *GNG*2, involved in monoamine signalling, particularly in postsynaptic signalling at serotonergic and dopaminergic synapses, was identified as a positional candidate gene in chicken pecking in a genome-wide analysis study [18]. These clues suggest that bird behaviour and pigment traits might be influenced by dopa analogues or other mid-products. However, further well-designed experiments are required to prove this hypothesis.

Meanwhile, interesting phenomena appeared in the comparisons among the mingled group and two pure colour groups (red or white groups). Compared with the white group hens (higher aggression level), hens in the mingled groups showed a significantly lower occurrence of aggressive behaviours. This might be explained by the existence of a “mixed-phenomenon” effect, which occurs in certain vertebrate species, e.g. fish schools, ungulate herds, and bird flocks. Mixed-species associations of birds are roving groups of individuals comprising at least two species searching for food together [38].

Considering these findings, we constructed a cross between WL and RIR, and all the progenies exhibited the same genotype on the *PMEL17* locus (*Ii*). We observed behavioural differences between the red- and white-feathered female progenies after they attained sexual maturity. Our results showed that feather colours could affect the behaviour independent of the *PMEL17* locus and remained latent till sexual maturity at 26 weeks. However, it is not clear which gene controlled the pigmentation or behaviour in this population. To investigate the genetic bases for the pigmented offspring of WL and the relationship between the above behaviour-related genes and feather colour, further research is warranted.

## Conclusions

In the present F1 cross population, white-feathered hens were more aggressive than the red ones were. Compared with raising white chickens together, mixing red and white chickens together can reduce the occurrence of aggressive behaviours. Moreover, these differences in behaviour and phenotype were not caused by *PMEL17*, *SOX10*, or *SLC45A2*, which provide a good model for behavioural research of individual birds with different feather colours. Taken together, the distinct behavioural findings observed in this study provide novel insights into the mechanisms underlying the association between phenotype and behaviour in the present population and other chicken breeds.

## Supporting information

S1 Table Counts of two aggressive behaviours in six groups (5 days). (DOCX)
