# Supplementary material for: Feather colour affects the aggressive behaviour of chickens with the same genotype on the dominant white (I) locus
Source: PLoS One. 2019 May 2;14(5):e0215921. doi: 10.1371/journal.pone.0215921 (PMC6497237; doi:10.1371/journal.pone.0215921)
Supplement: S1 Table — (DOCX) [file pone.0215921.s001.docx]

Table S1. Counts of two aggressive behaviours in six groups (5 days).

| Group | Behaviour | Attacker | Victim | 2016/8/20 | | | 2016/8/21 | | | 2016/8/22 | | | 2016/8/23 | | | 2016/8/24 | | |
| --- | --- | --- | --- | --- | --- | --- | --- | --- | --- | --- | --- | --- | --- | --- | --- | --- | --- | --- |
|  |  |  |  | 12:00 - 12:10 | 12:10 - 12:20 | 12:20 - 12:30 | 12:00 - 12:10 | 12:10 - 12:20 | 12:20 - 12:30 | 12:00 - 12:10 | 12:10 - 12:20 | 12:20 - 12:30 | 12:00 - 12:10 | 12:10 - 12:20 | 12:20 - 12:30 | 12:00 - 12:10 | 12:10 - 12:20 | 12:20 - 12:30 |
| Red Group 1 | chase, attack | Red | Red | 1 | 11 | 8 | 8 | 5 | 2 | 18 | 18 | 15 | 6 | 7 | 13 | 6 | 4 | 5 |
|  | peck, threat | Red | Red | 24 | 54 | 53 | 33 | 25 | 22 | 69 | 35 | 69 | 53 | 67 | 65 | 48 | 44 | 60 |
| Red Group 2 | chase, attack | Red | Red | 12 | 5 | 6 | 3 | 2 | 0 | 10 | 17 | 13 | 16 | 5 | 10 | 13 | 3 | 9 |
|  | peck, threat | Red | Red | 55 | 31 | 36 | 38 | 51 | 48 | 32 | 47 | 47 | 62 | 51 | 64 | 90 | 70 | 70 |
| Mingle Group 1 | chase, attack | White | White | 1 | 1 | 3 | 2 | 2 | 1 | 0 | 1 | 4 | 0 | 0 | 2 | 4 | 4 | 2 |
|  |  | White | Red | 1 | 1 | 4 | 0 | 1 | 1 | 3 | 2 | 1 | 3 | 6 | 3 | 3 | 1 | 3 |
|  |  | Red | White | 1 | 2 | 4 | 1 | 4 | 5 | 2 | 0 | 5 | 5 | 3 | 5 | 7 | 9 | 2 |
|  |  | Red | Red | 1 | 5 | 3 | 3 | 2 | 3 | 1 | 1 | 4 | 4 | 1 | 8 | 7 | 0 | 3 |
|  | peck, threat | White | White | 9 | 8 | 8 | 8 | 4 | 3 | 9 | 1 | 6 | 4 | 5 | 7 | 3 | 7 | 5 |
|  |  | White | Red | 7 | 10 | 12 | 5 | 4 | 5 | 9 | 12 | 12 | 7 | 3 | 8 | 11 | 4 | 2 |
|  |  | Red | White | 16 | 24 | 11 | 3 | 6 | 6 | 16 | 15 | 13 | 11 | 5 | 9 | 29 | 9 | 10 |
|  |  | Red | Red | 15 | 20 | 10 | 7 | 6 | 9 | 13 | 13 | 10 | 15 | 5 | 8 | 22 | 2 | 11 |
| Mingle Group 2 | chase, attack | White | White | 2 | 0 | 7 | 3 | 3 | 1 | 2 | 8 | 6 | 2 | 3 | 2 | 1 | 7 | 6 |
|  |  | White | Red | 1 | 2 | 4 | 2 | 0 | 1 | 4 | 4 | 4 | 3 | 1 | 1 | 0 | 4 | 3 |
|  |  | Red | White | 1 | 9 | 1 | 3 | 0 | 2 | 1 | 1 | 1 | 4 | 2 | 1 | 4 | 3 | 1 |
|  |  | Red | Red | 1 | 1 | 1 | 1 | 1 | 0 | 2 | 0 | 3 | 1 | 0 | 0 | 1 | 1 | 0 |
|  | peck, threat | White | White | 10 | 4 | 18 | 5 | 5 | 15 | 15 | 14 | 15 | 7 | 6 | 3 | 12 | 12 | 11 |
|  |  | White | Red | 8 | 18 | 11 | 14 | 9 | 15 | 16 | 20 | 15 | 11 | 7 | 13 | 9 | 11 | 19 |
|  |  | Red | White | 4 | 21 | 13 | 7 | 7 | 12 | 8 | 7 | 10 | 5 | 7 | 6 | 6 | 9 | 7 |
|  |  | Red | Red | 6 | 11 | 13 | 8 | 6 | 20 | 9 | 10 | 12 | 7 | 11 | 9 | 3 | 4 | 8 |
| White Group 1 | chase, attack | White | White | 29 | 12 | 18 | 20 | 24 | 13 | 17 | 15 | 34 | 26 | 26 | 40 | 6 | 10 | 16 |
|  | peck, threat | White | White | 103 | 132 | 62 | 43 | 41 | 54 | 90 | 78 | 90 | 74 | 82 | 96 | 111 | 93 | 64 |
| White Group 2 | chase, attack | White | White | 19 | 16 | 15 | 4 | 16 | 15 | 16 | 15 | 14 | 13 | 15 | 11 | 13 | 11 | 10 |
|  | peck, threat | White | White | 84 | 60 | 104 | 57 | 56 | 77 | 53 | 69 | 92 | 77 | 93 | 94 | 101 | 96 | 70 |
